# Therapeutic Efficacy of a Novel Acetylated Tetrapeptide in Animal Models of Age-Related Macular Degeneration

**DOI:** 10.3390/ijms22083893

**Published:** 2021-04-09

**Authors:** Hye Cheong Koo, Yi-Yong Baek, Jun-Sup Choi, Young-Myeong Kim, Bokyung Sung, Min-Jung Kim, Jae Gyu Kim, Ji Chang You

**Affiliations:** 1 Avixgen Inc., Seoul 06649, Korea; koohj99@avixgen.com (H.C.K.); yybaek@avixgen.com (Y.-Y.B.); mjschoi@daum.net (J.-S.C.); bsung@avixgen.com (B.S.); mjkim208@avixgen.com (M.-J.K.); jagkim@avixgen.com (J.G.K.); 2 Department of Molecular and Cellular Biochemistry, School of Medicine, Kangwon National University, Chuncheon 24341, Gangwon-do, Korea; ymkim@kangwon.ac.kr; 3 National Research Laboratory for Molecular Virology, Department of Pathology, School of Medicine, The Catholic University of Korea, Seoul 06591, Korea

**Keywords:** acetylated tetrapeptide (Ac-RLYE), neovascular age-related macular degeneration, resistance, retinal neovascularization, laser-induced CNV model, VEGF, VEGFR-2

## Abstract

It has been shown previously that a novel tetrapeptide, Arg-Leu-Tyr-Glu (RLYE), derived from human plasminogen inhibits vascular endothelial growth factor (VEGF)-induced angiogenesis, suppresses choroidal neovascularization in mice by an inhibition of VEGF receptor-2 (VEGFR-2) specific signaling pathway. In this study, we report that a modified tetrapeptide (Ac-RLYE) showed improved anti-choroidal neovascularization (CNV) efficacy in a number of animal models of neovascular age-related macular degeneration (AMD) which include rat, rabbit, and minipig. The preventive and therapeutic in vivo efficacy of Ac-RLYE via following intravitreal administration was determined to be either similar or superior to that of ranibizumab and aflibercept. Assessment of the intraocular pharmacokinetic and toxicokinetic properties of Ac-RLYE in rabbits demonstrated that it rapidly reached the retina with minimal systemic exposure after a single intravitreal dose, and it did not accumulate in plasma during repetitive dosing (bi-weekly for 14 weeks). Our results suggested that Ac-RLYE has a great potential for an alternative therapeutics for neovascular (wet) AMD. Since the amino acids in human VEGFR-2 targeted by Ac-RLYE are conserved among the animals employed in this study, the therapeutic efficacies of Ac-RLYE evaluated in those animals are predicted to be observed in human patients suffering from retinal degenerative diseases.

## 1. Introduction

Age-related macular degeneration (AMD) causes blindness in senile populations aged 50 years and older. It is the third most common cause of blindness worldwide after cataract and glaucoma [1,2,3]. The prevalence of AMD is gradually growing, and neovascular AMD could affect 288 million people by 2040 globally [4].

There are largely two AMD types: Atrophic (dry) and neovascular (wet). Atrophic AMD is triggered by abnormalities in the retinal pigment epithelium–Bruch’s membrane–choriocapillaris complex and accounts for approximately 90% of all AMD cases. In neovascular AMD, which accounts for 10% of the AMD cases, new blood vessels appear in the choroid layer beneath the retina. It shows symptoms, such as retinal pigment epithelial tear, sensory retinal detachment, and retinal pigment epithelial tears, and may also affect central vision, resulting in blindness 2 months to 3 years after disease onset [5,6,7].

Treatment modalities for choroidal neovascularization (CNV) in AMD include laser photocoagulation, photodynamic therapy (PDT), and anti-vascular endothelial growth factor (VEGF) therapy. However, laser photocoagulation applied in treating CNV may cause post-treatment vision deterioration by damaging normal visual cells. PDT requires repeated treatment and has limited effects on minimally classic and occult CNV [8,9]. As a common therapy, anti-VEGF antibody treatment through direct intravitreal injection inhibits abnormally increased VEGF involved in the generation and growth of new blood vessels [10,11,12]. Ranibizumab, an anti-VEGF therapy, improves vision by effectively inhibiting CNV and decreasing leakage [13], but it represents a great patient burden due to its relatively high production cost compared with small molecules and synthetic peptides. Associated side effects reportedly include anemia, myalgia, retinal pigment epithelial tears, cataract, and intraocular inflammation, which are known to be triggered mainly by the administration route, a method of intravitreal injection [14]. However, since there is a conflicting debate on the function of VEGFR-1 in eye diseases, those side effects may also be attributable to the antibody’s mechanism of action (MOA) based on capturing VEGF, resulting in inhibition of VEGF receptor-1 (VEGFR-1) signaling [15,16,17,18]. Aflibercept, a fusion protein containing ligand-binding elements derived from the extracellular components of VEGFR-1 and VEGFR-2 fused to the Fc portion of IgG, has a similar MOA and the same limitations as ranibizumab [19,20,21].

Although anti-VEGF therapy has been commercialized, persistent intraretinal or subretinal fluid or recurrent exudation still occurs, and several reports indicate the occurrence of resistance to anti-VEGF agents. The need for developing other drugs, which can restore normal retinal vascular permeability and thus can be used as an alternative therapy or combination therapy, has been published [22]. For example, one study found that 81% of patients with resistance to bevacizumab and ranibizumab due to tachyphylaxis responded to treatment by switching these drugs to ranibizumab and bevacizumab, respectively [23]. In addition, retinal edema and pigment epithelial detachment regressed significantly after switching to aflibercept in patients insufficiently responding to prior therapy with ranibizumab or bevacizumab [24].

The VEGF-A/VEGFR-2 signaling pathway reportedly functions as the main axis of driving angiogenesis under physiological and pathological conditions [25], which inspired the development of VEGFR-2 inhibitors as alternative drug candidates for neovascular AMD [26,27,28] or retinopathy [29]. The novel therapies that inhibit VEGF-A/VEGFR-2 signaling are expected to treat neovascular AMD with effective and long-acting efficacies, and they should minimize the risk of possible side effects caused by inhibiting VEGFR-1 signaling. Moreover, similar to the switching between different anti-VEGF antibody drugs, the new VEGFR-2 inhibitors can be administered as alternative therapeutics in patients experiencing recurrent or refractory neovascular AMD. As one of the strategies for solving those unmet medical needs, an antiangiogenic tetrapeptide Arg-Leu-Tyr-Glu (RLYE) derived and engineered from the human plasminogen kringle-5 domain has been previously developed and shown to inhibit specifically the VEGFR-2 signaling pathway, which was confirmed by its ability to suppress CNV and tumor angiogenesis in appropriate mouse models [30,31,32] Moreover, RLYE optimization for improving serum stability was achieved by N-terminal acetylation, which produced Ac-RLYE that was even better in inhibiting tumor angiogenesis effectively in an animal model [33]. Here, we evaluated the anti-CNV efficacy of Ac-RLYE and its MOA in several animal models of wet AMD. We also assessed the intraocular PK properties of Ac-RLYE in rabbits treated with a single intravitreal dose, along with the drug’s TK properties in rabbits receiving a repeated intravitreal dose bi-weekly for 14 weeks. The Ac-RLYE profile based on our results suggested that this novel VEGF-2-specific antagonist appears to be a promising drug candidate for the treatment of neovascular (wet) AMD caused by pathological angiogenesis.

## 2. Results

### 2.1. Assessment of Antiangiogenic Activity of RLYE, R_(D)_LYE, and Ac-RLYE in Comparison with Aflibercept in a Mouse CNV Model

**Figure 1:**
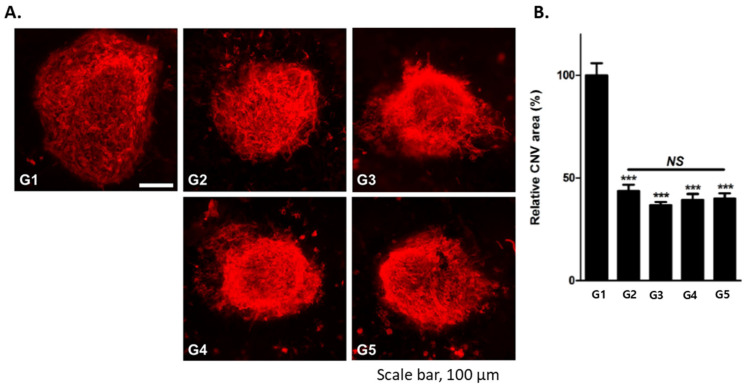
The effects of the intravitreal administration of RLYE, the modified RLYE variants [R_(D)_LYE and Ac-RLYE], and aflibercept on the area of choroidal neovascularization (CNV) in laser-induced CNV mouse models with treatment initiation on the same day as laser irradiation. (**A**): Representative fundus photography of retinal angiography analyzed by fluorescence microscope on day 7 after laser irradiation. (**B**): A quantitative image analysis results for the area of CNV using the ImageJ software (*n* = 6 mice per group). Data are expressed as mean ± S.D. G1: Vehicle control, G2: RLYE 1 ng/1 µL/eye, G3: R_(D)_LYE 1 ng/1 µL/eye, G4: Ac-RLYE 1 ng/1 µL/eye, G5: Aflibercept 145 ng/1 µL/eye. *** A significant difference at *p* < 0.001 level compared to the G1. NS: Non-significant difference. The intravitreal administration of RLYE (G2), R_(D)_LYE (G3), Ac-RLYE (G4), and aflibercept (G5) significantly reduced the area of CNV compared to vehicle group (arbitrarily set at 100%, G1). Treatment efficacy did not vary significantly between test and positive control drugs.

The serum half-life of Ac-RLYE (8.8 h) and R_(D)_LYE (7.0 h), in which L-Arg was replaced with D-Arg, was substantially improved compared with that of RLYE (1.2 h) in our previous study [33]. In the present study, we evaluated the effect of Ac-RLYE on the reduction of the CNV area in mice with laser-induced CNV, as described previously [34]. Each peptide and aflibercept used as the positive control were intravitreally injected on the same day as the laser irradiation. In comparison with a vehicle group (G1), the intravitreal administration of RLYE (G2), R_(D)_LYE (G3), Ac-RLYE (G4), and aflibercept (G5) significantly reduced the CNV area on day 7 after laser irradiation and test substance administration (Figure 1). The result confirmed the comparable antiangiogenic efficacy of Ac-RLYE, R_(D)_LYE, and RLYE in our mouse AMD model.

### 2.2. Comparison of Antiangiogenic Activity of Ac-RLYE and RLYE in a Diabetic Mouse Model

**Figure 2:**
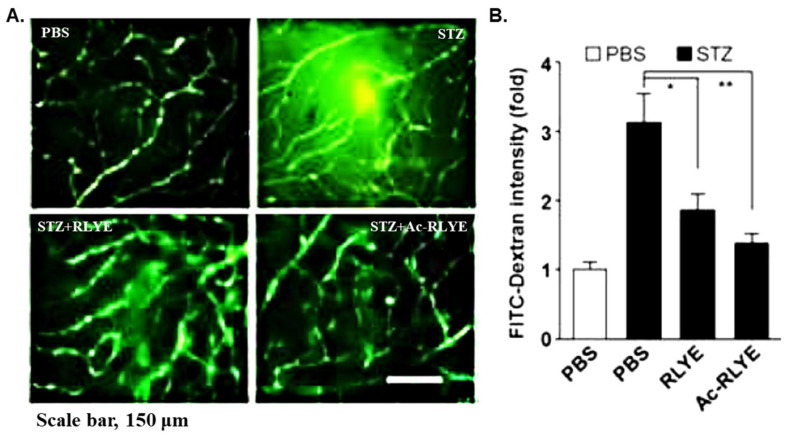
The effects of the intravitreal administration of RLYE and Ac-RLYE on inhibition of retinal vascular leakage in streptozotocin (STZ) induced diabetic mouse models. (**A**): Representative image of a retinal flat mount observed under a confocal microscope following injection of FITC-dextran into the left ventricle at 24 h after the substance treatment, (**B**): Measurement of the fluorescence of FITC-dextran leaked from retinal vessels by Fluoview software (*n* = 4 mice per group). The retinal vascular leakage increased in STZ-induced diabetic retinopathy mice was significantly inhibited by RLYE (*, 0.01 < *p* < 0.05) and Ac-RLYE (**, *p* < 0.01).

Diabetic retinopathy (DR) is a degenerative disease leading to blindness in the working-age population, and non-proliferative DR can progress to proliferative DR with abnormal neovascularization and vascular leakage mainly caused by increased production of VEGFs in the vitreous [35,36,37,38,39]. We compared the anti-DR efficacy of Ac-RLYE and RLYE in mice with DR induced by streptozotocin treatment administered 2 weeks prior to the peptide treatment. Specifically, the retinal vascular leakage increased within 2 weeks after streptozotocin induction, but then, the administration of RLYE (0.01 < *p* < 0.05) or Ac-RLYE (*p* < 0.01) effectively inhibited the leakage compared with that after administering vehicle (Figure 2). The result suggested that the inhibitory effect of Ac-RLYE on retinal vascular leakage was either equal or slightly higher than that of RLYE. Thus, Ac-RLYE also appeared to have efficacy as a therapeutic for DR.

### 2.3. Antiangiogenic Activity of Ac-RLYE in Animal Models of 2-Week Laser-Induced CNV

#### 2.3.1. Rat 2-Week CNV Model

Firstly, we wanted to confirm the retinal antiangiogenic activity of Ac-RLYE in a rat model of 2-week laser-induced CNV and compare the peptide’s efficacy with that of the positive control drug aflibercept. The test was conducted by administering either Ac-RLYE or aflibercept once to the rats on the same day as the laser irradiation.

**Figure 3:**
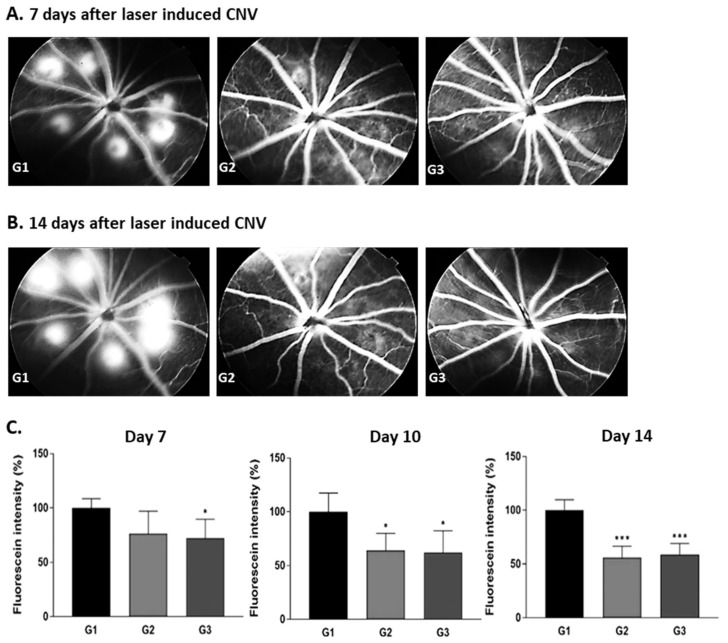
The effects of the intravitreal administration of Ac-RLYE and aflibercept on inhibition of choroidal neovascularization (CNV) in laser-induced CNV rat models with treatment of peptide or aflibercept at the same day as laser irradiation. The intensity of laser irradiation and spot size was empirically set to maintain the retinal fluorescence intensity in the vehicle treatment group at 2 weeks after laser irradiation. (**A**,**B**): Representative retinal fluorescence fundus image. (**C**): A quantitative image analysis result for retinal fluorescence intensity using the ImageJ software (*n* = 6 rats per group). The day of laser irradiation was designated as day 0. Data are expressed as mean ± S.D. G1: Vehicle control, G2: Aflibercept 400 µg/10 µL/eye, G3: Ac-RLYE 20 µg/50 µL/eye. ***/* A significant difference at *p* < 0.001/ *p* < 0.05 level compared to the G1. Compared to vehicle group (arbitrarily set at 100%, G1), the retinal fluorescence intensity of Ac-RLYE treated group (G3) was significantly different from day 7 after laser irradiation, whereas that of the aflibercept treated group (G2) was significantly decreased from day 10 after laser irradiation.

The retinal fluorescein intensity in the Ac-RLYE-treated group (G3) was significantly lower than that in vehicle control group (G1) on days 7 (*p* < 0.05), 10 (*p* < 0.05), and 14 (*p* < 0.001) after the laser irradiation. The retinal fluorescein intensity in the aflibercept-treated group (G2) was significantly lower than that in the vehicle control group (G1) on days 10 (*p* < 0.05) and 14 (*p* < 0.001) after the laser irradiation. The results (Figure 3) indicated that the anti-CNV efficacy of Ac-RLYE was similar to that of aflibercept. Moreover, in this model, the time to onset of the treatment effect was 3 days shorter in the Ac-RLYE treatment group than in the positive control drug group.

#### 2.3.2. Rabbit 2-Week CNV Model

**Figure 4:**
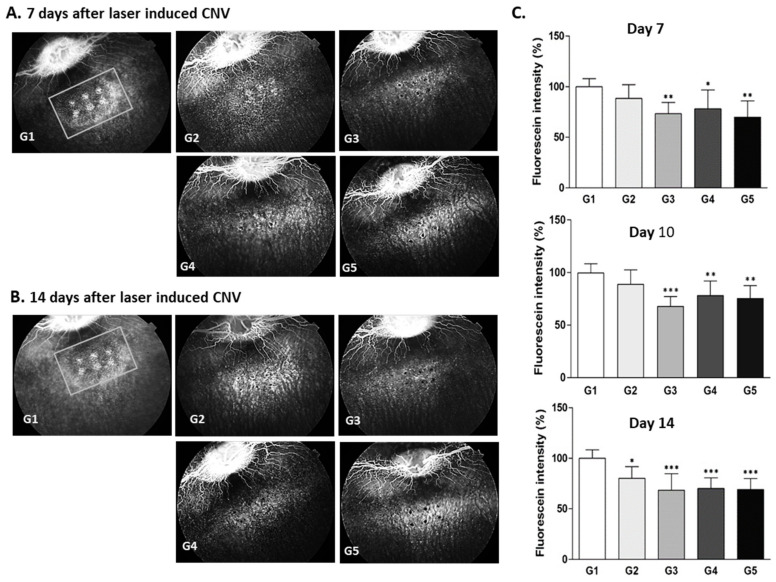
The effects of the intravitreal administration of Ac-RLYE and ranibizumab on inhibition of choroidal neovascularization (CNV) in laser-induced CNV rabbit models with treatment of peptide or ranibizumab at the same day as laser irradiation. The intensity of laser irradiation and spot size was empirically set to maintain the retinal fluorescence intensity in the vehicle treatment group at 2 weeks after laser irradiation. (**A**,**B**): Representative retinal fluorescence fundus image analyzed after injection of fluorescein sodium salt through the ear vein at each day after laser irradiation. (**C**): A quantitative image analysis result for retinal fluorescence intensity using the ImageJ software (*n* = 6 rabbits per group). The day of laser irradiation was designated as day 0. Data are expressed as mean ± S.D. G1: Vehicle control, G2: Ac-RLYE 0.2 µg/50 µL/eye, G3: Ac-RLYE 2 µg/50 µL/eye, G4: Ac-RLYE 20 µg/50 µL/eye, G5: Ranibizumab 500 µg/50 µL/eye. ***/**/* A significant difference at *p* < 0.001/*p* < 0.01/*p* < 0.05 level compared to the G1. Compared to the vehicle group (arbitrarily set at 100%, G1), the retinal fluorescence intensity in the Ac-RLYE groups was significantly decreased with a dose-dependent manner (G2–G4).

Secondly, the retinal antiangiogenic activity of Ac-RLYE was evaluated in a rabbit 2-week laser-induced CNV model in comparison with another positive control drug, ranibizumab. The test drug group (G3) treated with Ac-RLYE 2 μg/eye had a significantly lower retinal fluorescein intensity than the vehicle control group (G1) (*p* < 0.05) on day 3 after laser irradiation (data not shown). On days 7 and 10 after laser irradiation, the retinal fluorescein intensity was equal among two test drug groups (G3, G4) treated with Ac-RLYE 2, 20 μg/eye and the control drug group (G5) treated with ranibizumab 500 μg/eye, but significantly lower than that in the vehicle control group. Furthermore, the retinal fluorescein intensity in all three test drug groups (G2–G4) treated with Ac-RLYE 0.2, 2, 20 μg/eye and the control drug group (G5) treated with ranibizumab 500 μg/eye was significantly lower than that in the vehicle control group (*p* < 0.001 or *p* < 0.05) on day 14 after laser irradiation. These results (Figure 4) demonstrated that the significant anti-CNV effect of Ac-RLYE was dose-dependent within the dose range of 0.2–20 μg/eye. In addition, we found that the anti-AMD efficacy of 2 μg of Ac-RLYE (G3) was comparable to that of the positive control, ranibizumab, in this rabbit CNV model.

#### 2.3.3. Minipig 2-Week CNV Model

As the eye tissue and blood vessels of the minipig are very similar to that in human, we used the minipig 2-week laser-induced CNV model to evaluate the retinal antiangiogenic activity of Ac-RLYE in comparison with that of the positive control drug ranibizumab.

**Figure 5:**
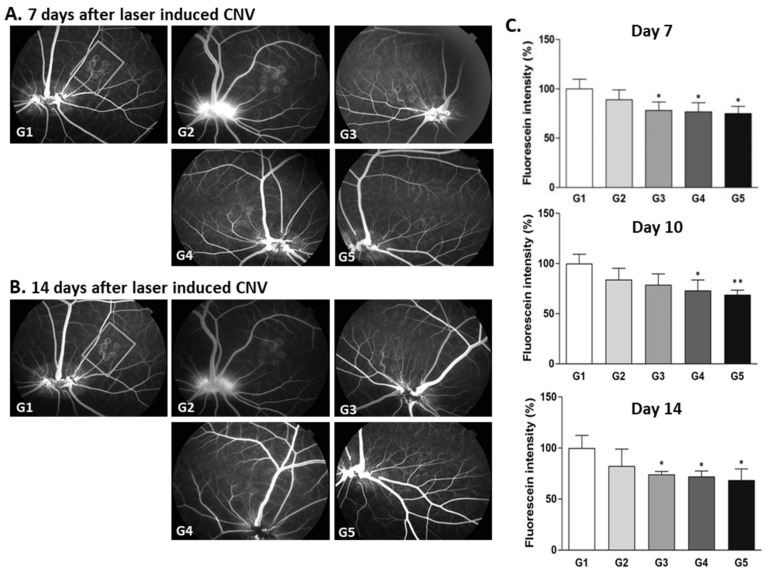
The effects of the intravitreal administration of Ac-RLYE and ranibizumab on inhibition of choroidal neovascularization (CNV) in laser-induced CNV minipig models with treatment peptide or ranibizumab at the same day as laser irradiation. The intensity of laser irradiation and spot size was empirically set to maintain the retinal fluorescence intensity in the vehicle treatment group at 2 weeks after laser irradiation. (**A**,**B**): Representative retinal fluorescence fundus image. (**C**): A quantitative image analysis result for retinal fluorescence intensity using the ImageJ software. The day of laser irradiation was designated as day 0. Data are expressed as mean ± S.D. G1: Vehicle control, G2: Ac-RLYE 1 µg/50 µL/eye, G3: Ac-RLYE 3 µg/50 µL/eye, G4: Ac-RLYE 10 µg/50 µL/eye, G5: Ranibizumab 500 µg/50 µL/eye (Both eyes of 2 minipigs per group were administrated). **/* A significant difference at *p* < 0.01/ *p* < 0.05 level compared to the G1. Compared to vehicle group (arbitrarily set at 100%, G1), the retinal fluorescence intensity in the Ac-RLYE groups was significantly decreased in a dose-dependent manner (G2–G4).

The test drug group (G3) treated with Ac-RLYE 3 μg/eye had a significantly lower retinal fluorescein intensity than the vehicle control group (G1) (*p* < 0.05) on days 7 and 14 after the laser irradiation. The test drug group (G4, *p* < 0.05) treated with Ac-RLYE 10 μg/eye and the control drug group (G5, *p* < 0.01 or *p* < 0.05) treated with ranibizumab 500 μg/eye had a significantly lower retinal fluorescein intensity than the vehicle control group (G1) on days 7, 10, and 14 after laser irradiation (Figure 5). The results demonstrated a significant dose-dependent anti-CNV effect of Ac-RLYE, and, more importantly, the inhibitory activity of the Ac-RLYE 10 μg/eye treatment (G4) was similar to that of the ranibizumab 500 μg/eye treatment (G5) in this minipig CNV model.

### 2.4. Determination of Antiangiogenic Activity of Ac-RLYE in Comparison with Ranibizumab and Aflibercept in a Rabbit 4-Week CNV Model 

We developed a rabbit 4-week CNV model to examine the anti-CNV effect of Ac-RLYE over an extended CNV period induced in rabbits using a higher laser irradiation intensity, which generated CNV lesions that were bigger than those obtained in the rabbit 2-week CNV model. The extended model maintained the CNV lesions even after 4 weeks after laser irradiation.

**Figure 6:**
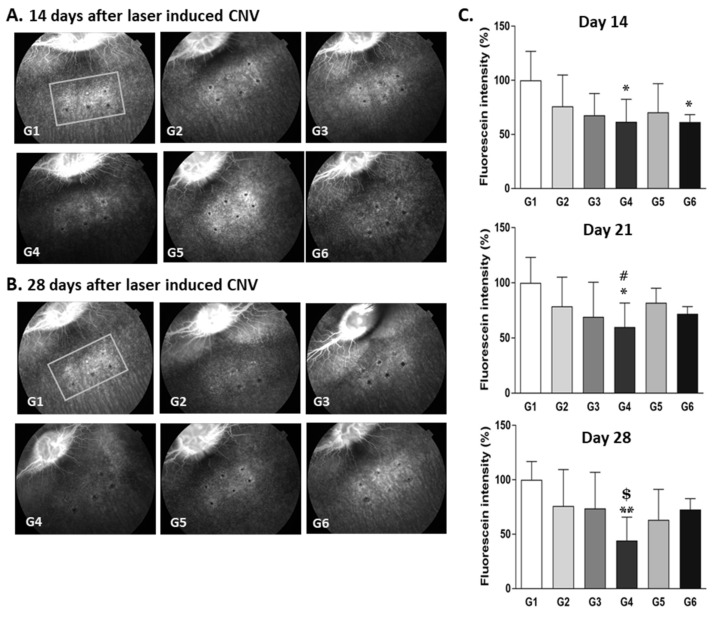
The effects of the intravitreal administration of Ac-RLYE, ranibizumab, and aflibercept on inhibition of choroidal neovascularization (CNV) in laser-induced CNV rabbit models with treatment of peptide or positive control at the same day as laser irradiation. The intensity of laser irradiation and spot size was empirically set in order to maintain the retinal fluorescence intensity in the vehicle treatment group at 4 weeks after laser irradiation. (**A**,**B**): Representative retinal fluorescence fundus image. (**C**): A quantitative image analysis result for retinal fluorescence intensity using the ImageJ software (*n* = 7 rabbits per group). The day of laser irradiation was designated as day 0. Data are expressed as mean ± S.D. G1: Vehicle control, G2: Ac-RLYE 2 µg/50 µL/eye, G3: Ac-RLYE 20 µg/50 µL/eye, G4: Ac-RLYE 100 µg/50 µL/eye, G5: Ranibizumab 500 µg/50 µL/eye, G6: aflibercept 2000 µg/50 µL/eye. **/* A significant difference at *p* < 0.01/*p* < 0.05 level compared to the G1. ^#^ A significant difference at *p* < 0.05 level compared to the G5^. $^ A significant difference at *p* < 0.05 level compared to the G6. Compared to vehicle group (arbitrarily set at 100%, G1), the retinal fluorescence intensity in the Ac-RLYE groups was significantly decreased in a dose-dependent manner (G3, G4). At 21 and 28th days after administration of the test substance, retinal fluorescence intensity of the Ac-RLYE 100 μg/eye treated group (G4) was significantly lower than that of the ranibizumab treated group (G5) or aflibercept treated group (G6), which suggest a superiority of Ac-RLYE over ranibizumab and aflibercept under this experiment condition.

In this model, which compared Ac-RLYE with the positive control drugs ranibizumab and aflibercept, the retinal fluorescence intensity tended to be substantially decreased by the Ac-RLYE treatment (G2–G4 treated with 2, 20, and 100 μg/eye, respectively) in a dose-dependent manner compared with that in the vehicle group (G1). Specifically, the test drug group (G4) treated with Ac-RLYE 100 μg/eye maintained a significantly lower retinal fluorescein intensity than the vehicle control group (G1) throughout the entire experiment of 4 weeks with induced CNV lesions. In contrast, the control drug group (G6) treated with aflibercept 2000 μg/eye maintained a significantly reduced retinal fluorescence intensity only until day 14 after CNV induction compared with that in the vehicle group (G1) in this extended rabbit CNV model. The Ac-RLYE-treated group (G4: 100 μg/eye) and the aflibercept-treated group (G6: 2000 μg/eye) had a significantly lower retinal fluorescein intensity than the vehicle control group (G1; *p* < 0.05) on day 14 after laser irradiation. Moreover, Ac-RLYE (G4: 100 μg/eye) had a significantly higher anti-AMD effect than the vehicle control (G1, *p* < 0.05) and ranibizumab (G5: 500 μg/eye; ^#^, *p* < 0.05) on day 21 and was also more effective than the vehicle control (G1; *p* < 0.01) and aflibercept (G4: 2000 μg/eye; ^$^, *p* < 0.05) on day 28 after laser irradiation (Figure 6). These results demonstrated the superiority of Ac-RLYE over ranibizumab and aflibercept as an anti-CNV treatment in this rabbit model. The higher and longer-lasting anti-CNV activity of Ac-RLYE compared with that of aflibercept and ranibizumab in this experiment could be related to the different drug types. Firstly, the difference in the MOA might be one reason: Aflibercept and ranibizumab capture soluble VEGF, which increases in the retinal tissue under pathological conditions, whereas Ac-RLYE specifically binds to VEGFR-2, inhibiting its phosphorylation and VEGF-A/VEGFR-2 signaling, which is the main axis driving angiogenesis. Secondly, even if the amount of Ac-RLYE (100 μg) used for treatment is small, the Ac-RLYE dose is 7.8 times higher than the aflibercept dose and 15.6 times higher than the ranibizumab dose when comparing the administered amounts based on moles due to Ac-RLYE’s low molecular weight.

### 2.5. Determination of Therapeutic Efficacies of Ac-RLYE in Animal CNV Model

We also wanted to compare the improved anti-CNV therapeutic efficacy of Ac-RLYE with that of RLYE and EYLR (the reverse form of the RLYE peptide) in an animal disease model. The peptides were administrated in mice with laser-induced CNV 10 days after the laser irradiation [32]. On day 4 after peptide treatment (14 days after the laser irradiation), EYLR did not affect the occurrence of CNV. However, the CNV was effectively reduced when treated with RLYE or Ac-RLYE, and Ac-RLYE was substantially more effective than RLYE (Supplementary Figure S1), suggesting that Ac-RLYE can be effectively used as a therapeutic agent for AMD-related angiogenesis.

To assess further the therapeutic efficacy of Ac-RLYE in a more relevant model, we established the rabbit 7-week CNV model by irradiating the rabbit retina with a laser and perform the test drug treatment on day 7 after laser irradiation, i.e., 1-week CNV induction before treatment initiation. The retinal antiangiogenic activity of Ac-RLYE measured as its effect on the retinal fluorescein intensity and CNV area was evaluated in comparison with a positive control aflibercept in that model.

**Figure 7:**
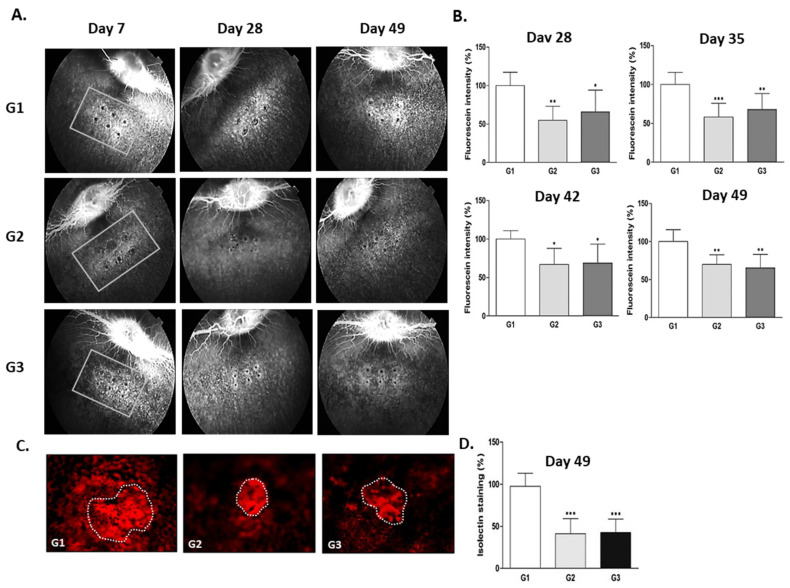
The therapeutic efficacies of the intravitreal administration of Ac-RLYE and aflibercept on choroidal neovascularization (CNV) in laser-induced CNV rabbit models with treatment of peptide or aflibercept 1 week after laser irradiation to induce active CNV for 1 week. The intensity of laser irradiation and spot size was empirically set in order to maintain the retinal fluorescence intensity in the vehicle treatment group at 7 weeks after laser irradiation. (**A**): Representative retinal fluorescence fundus image. (**B**): A quantitative image analysis result for retinal fluorescence intensity using the ImageJ software. (**C**): Representative fundus photography of retinal angiography (parts shown by a dotted line represent the boundary of a choroidal neovascularization membrane) analyzed by fluorescence microscope at Day 49 after laser induced CNV (Day 42 from the test substance treatment). (**D**): A quantitative image analysis result for the area of CNV using the ImageJ software. The day of laser irradiation was designated as day 0. Data are expressed as mean ± S.D. G1: Vehicle control, G2: Ac-RLYE 100 µg/50 µL/eye, G3: Aflibercept 2000 µg/50 µL/eye (*n* = 7 rabbits per group). ***/**/* A significant difference at *p* < 0.001/*p* < 0.01/*p* < 0.05 level compared to the G1. Compared to retinal fluorescence intensity observed in vehicle group (arbitrarily set at 100%, G1), the intravitreal administration of Ac-RLYE (G2) and aflibercept (G3) significantly reduced fluorescence intensity.

The control drug group (G3) treated with aflibercept 2000 μg/eye had a significantly lower retinal fluorescence intensity than the vehicle control group (G1, *p* < 0.01) on day 7 after test substance administration (day 14 after laser irradiation, data not shown). On days 14, 21, 28, 35, and 42 after test substance administration (which corresponded to days 21, 28, 35, 42, and 49 after laser irradiation), the test drug group (G2) treated with Ac-RLYE 100 μg/eye and the control drug group (G3) treated with aflibercept 2000 μg/eye had a significantly lower retinal fluorescence intensity than the vehicle control group (*p* < 0.001, *p* < 0.01, or *p* < 0.05, respectively) (Figure 7A,B), demonstrating that Ac-RLYE could effectively treat CNV until day 42 after a single intravitreal dose (day 49 after laser irradiation). Quantification of the CNV area showed that Ac-RLYE 100 μg/eye and aflibercept 2000 μg/eye reduced the CNV lesions to 36.0% and 38.9%, respectively, compared with those in the vehicle control group on day 42 after drug administration (Figure 7C,D).

### 2.6. Determination of the Intraocular PK Properties of Ac-RLYE

The intraocular PK properties of Ac-RLYE were assessed in rabbits receiving intravitreally administrated Ac-RLYE (20, 100 μg/eye). The concentration of Ac-RLYE in rabbit plasma and ocular tissues, such as the vitreous body and retina, including the choroid and aqueous humor, was determined using LC-MS/MS. The following quantitative ranges were found: 0.1–50 ng/mL for retina, including the choroid and plasma; 0.01–10 μg/mL for vitreous body and aqueous humor.

No significant interfering peaks were observed at retention times of either analytes or the internal standard in LC-MS/MS chromatograms of blank rabbit plasma and ocular tissue samples. The correlation coefficient of Ac-RLYE was more than 0.98 in all cases.

The PK parameters of AUC, C_max_, T_max_, t_1/2_, distribution volume (V_z(terminal)_/F), and clearance (CL/F) calculated using the non-compartmental analysis model are summarized in Table 1. Following a single intravitreal administration of Ac-RLYE 20 μg/eye or 100 μg/eye, Ac-RLYE rapidly reached the retina, including the choroid, within 2 h, which is attributable to its low molecular weight (621 Da). Ac-RLYE was detectable until 12 h and 72 h, following treatment with 20 and 100 μg/eye, respectively, further confirming the potential of Ac-RLYE for clinical development as neovascular (wet) AMD therapy. Remarkably, a fast onset of clinical efficacy based on the improvement of best-corrected visual acuity within 3 days of Ac-RLYE injection to AMD patients has been observed lately in a clinical trial in Korea (unpublished data). Either Ac-RLYE dose, 20 μg/eye or 100 μg/eye, was detected until 72 h in the vitreous body and until 24 h in the aqueous humor. The t_1/2_ of Ac-RLYE was 8–9 h in the ocular compartments. In the plasma after administering at Ac-RLYE 100 μg/eye, the peptide was detectable until 12 h at trace level (0.1–0.8 ng/mL, which is less than 0.67% of vitreous exposure when comparing AUC_last_). Following a single intravitreal dose in rabbits, the increase in plasma concentration of Ac-RLYE (AUC and C_max_) was non-proportional, resulting in nonlinear kinetics with minimal systemic exposure; its exposures in ocular tissues (AUC_last_) increased nonlinearly within the dose range of 20–100 µg/eye. A steep, non-proportional increase of the Ac-RLYE concentration in the retina including in the choroid and aqueous humor, was observed after administering higher doses. However, the AUC value for two dose levels in the vitreous body displayed a flat, non-proportional increase of the Ac-RLYE concentration. This apparent disproportionate increase and decrease of the Ac-RLYE concentration in different ocular tissues were most probably due to an initial saturation associated with the administration route.

In 30 New Zealand White rabbits (5 rabbits per each 6 sampling point), the whole blood was collected from jugular vein and then ocular tissues (vitreous body, retina including the choroid, aqueous humor) were sampled from rabbits euthanized at the scheduled sampling points (before Ac-RLYE administration, 2, 6, 12, 24, 72 h following administration of Ac-RLYE 20 µg/eye and 100 µg/eye).

For the differences of apparent volume of distribution (Vz(terminal)/F) in the retina, including the choroid, it was estimated that the terminal elimination rate constant (λz) in high dose was higher than that in low dose. The rate of elimination varied significantly between the ocular tissues and plasma, but similar elimination rates were observed in the ocular tissues, suggesting that there was no significant difference in the rate of elimination among ocular tissues.

### 2.7. Determination of the TK Properties of Ac-RLYE

The intraocular TK properties of Ac-RLYE were assessed by repeated dosing. Rabbits were intravitreally dosed at 80, 180, and 400 μg/head, bi-weekly for 14 weeks. The TK parameters of AUC up to 14 days (AUC_0–14day_), C_max_, T_max_, and elimination half-life (t_1/2_) derived using a non-compartmental analysis model are summarized in Table 2. Following the 1st intravitreal administration of Ac-RLYE on day 1 or the 8th dose at week 14, the maximum plasma level was reached after 0.25–3 days (day 1) or 0.25–3 days (week 14). The Ac-RLYE t_1/2_ could not be determined due to insufficient data points in the elimination phase. The Ac-RLYE plasma level was generally cleared within one day post-dose, but was detected at the level of 0.070–0.605 ng/mL for up to 10 days post-dose in one male and two female rabbits receiving the first dose of 400 μg/head.

The whole blood was collected from jugular vein and then ocular tissues (vitreous body, retina including the choroid, aqueous humor) were sampled from New Zealand White rabbits (5 males and 5 females per each dose group) euthanized at the scheduled sampling points [before Ac-RLYE administration, 6, 12, 24 h, 2, 3, 5, 7, 10, and 14 days after intravitreal administration of Ac-RLYE 0, 40, 90, and 200 μg/eye (both eyes; 0, 80, 180, and 400 μg/head, respectively) bi-weekly for 14 weeks].

We then examined the dose proportionality. When the dose increased by 2.3-fold, AUC_0–14day_ and C_max_ increased by 0.71- and 2.3-fold (male) or 3.8- and 2.5-fold (female) on day 1, and by 2.2- and 3.2-fold (male) or 9.0- and 5.9-fold (female) in week 14, respectively. When the dose increased by 5-fold, AUC_0–14day_ and C_max_ increased by 1.7- and 4.6-fold (male) or 10.7- and 6.1-fold (female) on day 1, and by 7.4- and 8.7-fold (male) or 30.4- and 18.7-fold (female) in week 14. In general, systemic exposure to Ac-RLYE increased in a dose-related manner. We also assessed the effect of repeated dosing. The accumulation ratio (Week 14/Day 1) for AUC_0–14day_ and C_max_ following the 14-week repeated administration was in the range of 0.13–0.66 and 0.18–0.71, respectively. Thus, systemic exposure to Ac-RLYE relatively decreased with repeated dosing over 14 weeks, and did not show accumulation, which is beneficial for safety and should be considered for maintaining therapeutic efficacy by adjusting the dosing cycle possibly about once every 2–3 months.

## 3. Discussion

Anti-VEGF antibody therapies have been used in patients suffering from retinal degenerative diseases, but there are still unmet needs for alternative or combination therapy due to treatment failure or insufficient drug response and various side effects, such as anemia, myalgia, retinal pigment epithelial tears, cataract, elevated intraocular pressure, and intraocular inflammation [14]. When there is still persistent intraretinal or subretinal fluid or recurrent exudation during examination with optical coherence tomography even after 3 or more treatments with intravitreal anti-VEGF antibody, these patients are classified as refractory or resistant neovascular (wet) AMD groups and need to obtain switch therapy to other anti-VEGF drugs [22], resulting in improvement of best-corrected visual acuity and the regression of retinal edema and pigment epithelial detachment [23,24]. Although the adverse events occurring in anti-VEGF antibody therapies are mainly attributed to intravitreal injection [14], they could also be caused by depleting VEGF, leading to inhibition of VEGFR-1 signaling. As VEGFR-1 plays an important physiological role in normal hematopoiesis and the movement of red blood cells and immune cells, blocking its signaling pathway may have fatal side effects. Moreover, there have been findings on two opposite roles of VEGFR-1, such as disease exacerbation due to worsening inflammation, and disease recovery through retinal stabilization in retinal degenerative diseases [15,16,17,18]. Hence, there is an urgent need to develop new therapeutic agents with high efficacy for anti-retinal degenerative diseases and minimized side effects caused by the inhibition of VEGFR-1 signaling.

Accordingly, several chemical inhibitors acting via the VEGFR-2 signaling pathway have been sought and reported [40,41]. We also identified and reported a novel antiangiogenic peptide, RLYE derived from the human plasminogen kringle-5 domain to inhibit the VEGF-A/VEGFR-2 signaling pathway by specifically targeting VEGFR-2 at the same binding site in VEGF-A [31,42] as follows. Charged residues at both termini (Arg and Glu residues) of RLYE bind electrostatically to hydrophilic pockets, Glu140, and Lys286 of human VEGFR-2. The Leu side chain and the aromatic ring of Tyr in Ac-RLYE are closely positioned to a hydrophobic patch, Val216–218, in human VEGFR-2, facilitating hydrophobic interactions. In addition, the Arg residue and the hydroxyl group of Tyr also interact with Asn253 of human VEGFR-2 by forming hydrogen bonds. Based on those specific interactions with VEGFR-2, RLYE suppresses CNV and tumor angiogenesis in mice model [30,32]. Furthermore, Ac-RLYE and R_(D)_LYE were derived by modification for improving serum stability and achieving better activity against tumor angiogenesis in an appropriate animal model [33].

In the present study, based on the antiangiogenic activity of RLYE and its modified derivatives in previous studies [30,31,32,33], the anti-CNV efficacy of Ac-RLYE was evaluated further in several animal wet AMD models. When RLYE, the modified peptides, and aflibercept were applied in mouse CNV, a prevention model of neovascular AMD, on the same day of laser induction and analyzed on day 7 after laser induction, the anti-CNV effect of RLYE and the modified peptides, including Ac-RLYE, was similar to that of aflibercept based on the reduction of CNV area (Figure 1); this suggested that Ac-RLYE and the positive control aflibercept had a comparable anti-AMD activity. Based on that result, Ac-RLYE was further tested in more relevant models (rat, rabbit, and minipig) with longer observation periods (2–4 weeks). At the range of 0.2–100 μg/eye, the anti-CNV efficacy of Ac-RLYE was dose-dependent and, at the range of 2–20 μg/eye, comparable with the positive controls aflibercept or ranibizumab in animal models of 2-week CNV (Figure 3, Figure 4 and Figure 5). The preventive inhibitory effect of Ac-RLYE on AMD is similar to that of another peptide inhibitor (ATWLPPR) of VEGFR-2, and it can abolish VEGF-induced angiogenesis in a rabbit corneal model [43], in which slow-releasing implants of hydrogel containing bovine serum albumin, VEGF, and/or peptide were inserted into the corneal stroma, and anti-CNV effects were assessed on day 12.

In the rabbit 4-week CNV model, the extended laser-induced model developed for evaluating the differential preventive inhibitory effect of Ac-RLYE on AMD, aflibercept was effective by reducing the retinal fluorescence intensity until 2 weeks post-treatment in this rabbit model. On the other hand, the preventive anti-AMD efficacy of Ac-RLYE (100 μg/eye) was observed over the entire analysis period of 4 weeks post-treatment, and its activity was superior to ranibizumab or aflibercept with significantly greater reductions in retinal fluorescein intensity compared with the vehicle control (Figure 6), which might be related to the different MOA of Ac-RLYE and its low molecular weight, achieving higher doses based on mole. These results suggested the potential use of Ac-RLYE in switch therapy for patients suffering from recurrent or refractory neovascular AMD. Even though the MOA and the CNV induction method in animal wet AMD models might be a little different from those of Ac-RLYE, there have been reports that the efficacy doses of peptides on CNV inhibitory effect have been found to be 10~20 μg or more (for the case of CAD27 with calreticulin anti-angiogenesis mechanism of action in rat CNV model) [44] or 20 μM~2 mM (Z-VADFMK as a pan-caspase inhibitor, D-JNKi peptide as a JNK inhibitor in mouse CNV model) [45], so it can be considered that the efficacy dose of Ac-RLYE (at or above the dose of 20 μg or 32 μM) appears to be comparable to that of other peptide or protein inhibitors with anti-CNV activity.

To further determine the therapeutic efficacy of Ac-RLYE, we applied RLYE, the modified peptides, or aflibercept to mouse or rabbit CNV in a therapeutic model of neovascular AMD. Peptide treatment occurred 1 week (rabbit) or 10 days (mouse) after laser induction, and the follow-up analysis occurred 4 days (mouse) or 6 weeks (rabbit) after administration of each peptide. Compared with vehicle and EYLR (control peptide), treatment with RLYE and Ac-RLYE had a significant therapeutic anti-AMD effect based on the degree of CNV area reduction compared with vehicle, which was 75% (Ac-RLYE) or 55% (RLYE) in the mouse CNV model (Supplementary Figure S1). This was greater than the preventive anti-AMD effect, the reduction by 60% in the CNV area shown in Figure 1. It suggests that Ac-RLYE can be used as a therapeutic and preventive agent against AMD. In addition, the therapeutic effect of Ac-RLYE on AMD was comparable to that of aflibercept until 7 weeks after laser irradiation (6 weeks post-treatment) in the rabbit CNV model. The CNV area was reduced to 38.9% (Ac-RLYE) or 36% (aflibercept) compared with vehicle (Figure 7). These results were in line with the anti-CNV efficacy of ranibizumab and bevacizumab, even when they were applied to a therapeutic rabbit CNV model, in which CNV was induced by corneal suture, and the anti-VEGF antibodies were administrated on day 7 after suture placement, the degree of reduction of the CNV area compared with vehicle was 53%–60% (ranibizumab) or 46%–60% (bevacizumab) 1 week post-treatment [46]. 

We concluded that Ac-RLYE demonstrated a higher or comparable efficacy for AMD, considering the significantly lower effective dose of Ac-RLYE, compared with ranibizumab and aflibercept based on nonclinical efficacy studies using various preventive or therapeutic animal CNV or DR models.

The PK analysis determined that the t_1/2_ of Ac-RLYE was 8–9 h in the ocular compartments, which is shorter compared with that of ranibizumab (52.4 h) [47] and aflibercept (94.1 h) [48], but its apparent volume of distribution in the retina (including the choroid) was 846.31 L and 32.94 L at 20 and 100 μg/eye, respectively, which is much higher than that of ranibizumab (2.7 mL) [47] and aflibercept (1.4 mL) [48]. The different MOA of Ac-RLYE, which is based on the direct inhibition of the VEGF-VEFR-2 interaction rather than capturing soluble VEGF as the anti-VEGF antibody therapy, and the lower molecular weight and higher volume of distribution in ocular tissues can potentially contribute to the therapeutic efficacy of Ac-RLYE, which had a duration of 4–6 weeks at or above the dose of 20 μg/eye (intravitreal injection) in AMD animal models. Further studies may need to address the association between the duration of action of Ac-RLYE in the animal model of laser-induced CNV and the t_1/2_ and distribution profiles of Ac-RLYE in ocular tissues. On this regards, it could be useful to develop a biodegradable deliver system to inject intravitreally the Ac-RLYE, avoiding a multiple treatment [49].

The TK of Ac-RLYE was examined following repeated intravitreal administration of Ac-RLYE for 14 weeks in rabbits (8 times, once bi-weekly). There were no Ac-RLYE-related effects or abnormal findings in body weight, food consumption, hematological analysis, clinical signs, ophthalmological examination, clinical biochemistry, organ weight, necropsy findings, and histopathological examination. The total protein concentration decrease and the albumin/globulin (A/G) ratio increase in male rabbits of the 400 µg/head/day group were not considered to be related to Ac-RLYE because they were not accompanied by any morphological changes or clinical biochemical changes related to liver and/or kidney. In addition, there were no immune deficiency-related changes due to globulin decrease. Capsule rupture in lenses of males in the 180 and 400 µg/head/day groups and females in the vehicle and 400 µg/head/day groups were not considered to be related to Ac-RLYE because this change lacked a dose–response relationship and was observed in a vehicle group.

Human studies determining the antiangiogenic activity of intravitreal injection with Ac-RLYE are currently in progress as phase I clinical trials with AMD patients in South Korea with the initial result of safety and anti-AMD efficacy measured as improvement of visual acuity and the reduction of central macular thickness and intraretinal fluid for more than 12 weeks after one dose administration. Sequence alignment analysis show that the amino acids of the human VEGFR-2 involved in interactions with Ac-RLYE are conserved among the animals (mouse, pig, and rabbit) employed in this study, except for one amino acid in the rat sequence (Supplementary Figure S2). The rat VEGFR-2 amino acid targeted by the tyrosine of Ac-RLYE is leucine instead of valine, but both amino acids are hydrophobic, which may not affect the hydrophobic interaction with tyrosine. Therefore, we hypothesize that preventive or therapeutic efficacies of Ac-RLYE in retinal neovascularization or diabetic retinopathy determined in those animal models are likely to be observed in human patients, and we propose that nonclinical effects observed in the animal studies can be utilized to predict the inhibitory efficacy of Ac-RLYE on retinal angiogenesis and vascular hyperpermeability in patients suffering from retinal degenerative diseases. As RLYE also suppressed retinal endothelial permeability and CNV by directly inhibiting the VEGF-A/VEGFR-2 signaling pathway in a previous study [32], Ac-RLYE could have the potential to treat and diminish the occurrence of resistance to anti-VEGF therapies when used as combination therapy or alternative therapy.

## 4. Materials and Methods

### 4.1. Peptides

The peptides RLYE, Ac-RLYE, EYLR, and R_(D)_LYE were purchased from Peptron (Daejeon, Korea) or Chempeptide Limited (Shanghai, China).

### 4.2. Assessment of Antiangiogenic Activity of RLYE, R_(D)_LYE, and Ac-RLYE in Comparison with Aflibercept in a Mouse CNV Model

Six-week-old male C57BL/6J mice were purchased from Central Laboratory Animal and then maintained in a specific pathogen free facility in Seoul National University. After deep anesthesia with zolazepam plus tiletamine (3.75 mg/kg, Virbac, Seoul, Korea) and xylazine (7.5 mg/kg, Bayer AG, Leverkusen, Germany), mice were treated with a customized laser indirect ophthalmoscope system (ILOODA) to break Bruch’s membrane (300 μm spot size, 300 mW power, and 100 ms exposure time) as described previously [34]. After the laser irradiation, 10 μL PBS (vehicle), 1 ng of each peptide (RLYE, R_(D)_LYE and Ac-RLYE), or 145 ng of aflibercept was administered into the vitreous cavity of mice (six per each treatment group) at the same day as laser irradiation. At 7 days after the laser irradiation, the RPE-choroid-scleral complexes were prepared from the enucleated eyes. After immunostaining of the RPE-choroid-scleral complexes with isolectin B4 conjugated with Alexa Fluor-594 (1:100; cat. No. I21413, Invitrogen, Waltham, MA, USA), the CNV area was quantitatively analyzed by the measurement of the area using the ImageJ software. All animal experiments were carried out according to the guidelines of the Ethics Committee for Protection and Use of Experimental Animals of Seoul National University.

### 4.3. Comparison of Antiangiogenic Activity of RLYE and Ac-RLYE in Diabetic Mouse Model

To prepare a streptozotocin-induced diabetic mouse model, a freshly prepared STZ solution (100 mM) in a citrate buffer (100 mM, pH 4.5) was injected intraperitoneally at 150 mg/kg into the mice, and to prevent hypoglycemic shock, 10% sucrose was sufficiently provided. After two days, blood glucose was measured using an Accu-Chek Performa blood glucose meter (Roche Diagnostics GmbH, Mannheim, Germany), and when non-fasting blood glucose within 1 to 2 weeks was maintained at 300 mg/dl or more, the mice were used as diabetic animal models. The diabetes-induced mice were anesthetized with 2% avertin, and 1 μL of a 1.5 mM EYLR, RLYE, or Ac-RLYE solution was intravitreally injected (4 mice per each treatment group). Here, the same amount of PBS was injected into the control group. The peptide was injected, and after 24 h, 1 mL (1.25 mg) of FITC-dextran (250 kDa) was injected into the left ventricle of each mouse to allow circulation for approximately 5 min. The mice were euthanized by cervical dislocation, and the eyeballs were extracted and fixed in 4% paraformaldehyde at room temperature for 1 h. The retina was isolated from the fixed eyeball to prepare a retinal flat mount, and vascular leakage was observed under a confocal microscope. Meanwhile, the fluorescence of FITC-dextran leaked from retinal vessels was measured using Fluoview software.

All animal experiments were carried out according to the guidelines of the Ethics Committee for Protection and Use of Experimental Animals of Kangwon National University.

### 4.4. Antiangiogenic Activity of Ac-RLYE in Comparison with Ranibizumab or Aflibercept in Animal Models of 2-Week Laser-Induced CNV (Rat, Rabbit, Minipig)

#### 4.4.1. The Induction of Animal Models of 2-Week CNV (Rat, Rabbit, Minipig)

The following animals were used; Brown Norway rat (strains: BN/SsN Slc, 5 weeks old male) supplied by Central Lab. Animal Inc. (Seoul, Korea), Chinchilla rabbit (body weight of 2.0~2.5 kg, male) supplied by Dream Bio (Seoul, Korea), minipig (body weight of 17.0~21.0 kg, male) supplied by CRONEX Co., Ltd. (Hwaseong, Korea). Those animals were quarantined and acclimated for 7 days from the date of acquisition with the observation of clinical signs, check of health status. Healthy animals selected after the acclimation period were weighed and then, referring to the rank of body weight, distributed randomly so that each group has uniform body weight.

After instillation of 1% Midriacyl eye drop to right eye (rat, rabbit) or both eyes (minipig), animals were anesthetized with Zoletil 50 (Virbac, Seoul, Korea) and xylazine (Rompun^®^, Bayer AG, Leverkusen, Germany). After confirmation of anesthesia, laser irradiation (Elite, Lumenis, San Jose, CA, USA) was performed using a laser with a center wavelength of 532 nm, an incident power of 100~150 mW (100 mW for rat, 125 mW for rabbit, and 150 mW for minipig), spot size of 100~150 μm (100 μm for rat and rabbit, 150 μm for minipig) and a pulse duration of 0.1 s. The intensity of laser irradiation was empirically set in order to maintain the retinal fluorescence intensity in the vehicle treatment group of each animal model at 2 weeks after laser irradiation. Six spots were made at about 6 o’clock centered on optic nerve.

#### 4.4.2. Administration of Test Substance and CNV Measurement

After the animals were anesthetized, the test substance (50 μL of vehicle, Ac-RLYE or positive control) was administered once into the right eye of the animal using a syringe equipped with a 31-gaμge needle on the day of laser irradiation.

The administration groups per each animal are as follows; in rat CNV model, vehicle [phosphate buffered saline (PBS), pH 7.4] control group (G1), aflibercept 400 μg/10 μL/eye treated group (G2), and Ac-RLYE 20 μg/10 μL/eye treated group (G3) were composed (6 rats per group). Vehicle (PBS, pH 7.4) control group (G1), Ac-RLYE 0.2, 2, 20 μg/50 μL/eye treated group (G2–G4), and ranibizumab 500 μg/50 μL/eye treated group (G5) were composed in rabbit CNV model (6 rabbits per group). Vehicle (PBS, pH 7.4) control group (G1), Ac-RLYE 1, 3, 10 μg/50 μL/eye treated group (G2–G4), and ranibizumab 500 μg/50 μL/eye treated group (G5) were composed in minipig CNV model [2 minipigs (both eyes) per group].

From the day of laser irradiation to 3, 7, 10, and 14 days after laser induced CNV, the animals were instilled with a 1% Mydriacyl eye drop in the right eye (rat, rabbit) or both eyes (minipig), and then anesthesia was performed by Zoletil 50 (Virbac, Seoul, Korea) and xylazine (Rompun^®^, Bayer AG, Leverkusen, Germany). Approximately 1 mL of 2% fluorescein sodium salt solution was injected through the ear vein and photographs were taken within 2 min using a fundus camera (TRC-50IX, TOPCON, Tokyo, Japan). Retinal CNV confirmation and efficacy evaluation were performed using retinal fluorescence fundus photographs. Image analysis was performed by using ImageJ software.

All animal experiments were carried out according to the guidelines of the Ethics Committee for Protection and Use of Experimental Animals of KNOTUS Co., Ltd. (Incheon, Korea) [Approval number: KNOTUS Institutional Animal Care and Use Committee (IACUC) 19-KE-024, 16-KE-197 and 16-KE-262].

### 4.5. Antiangiogenic Activity of Ac-RLYE in Comparison with Ranibizumab and Aflibercept in a Rabbit 4-Week CNV Model

#### 4.5.1. The Induction of a Rabbit 4-Week CNV Model

After healthy Chinchilla rabbits selected after the acclimation period were instilled with a 1% Midracyl eye drop to the right eye and anesthetized, laser irradiation was performed using a laser with a center wavelength of 532 nm, an incident power of 150 mW, spot size of 200 μm, and pulse duration of 0.1 sec. The intensity of laser irradiation was empirically set to be higher than that used in the rabbit 2-week CNV model in order to maintain the retinal fluorescence intensity in the vehicle treatment group at 4 weeks after laser irradiation. Six spots were made at about 6 o’clock centered on the optic nerve.

#### 4.5.2. Administration of Test Substance and CNV Measurement

After the animals were anesthetized, the test substance (50 μL of vehicle, Ac-RLYE or positive control) was administered once into the right eye using a syringe equipped with a 31-gauge needle with the following administration groups (7 rabbits per group): Vehicle (PBS, pH 7.4) control group (G1), Ac-RLYE 2, 20, 100 μg/50 μL/eye treated group (G2–G4), ranibizumab 500 μg/50 μL/eye treated group (G5), and aflibercept 2000 μg/50 μL/eye treated group (G6).

From the day of laser irradiation to 7, 14, 21, and 28 days after laser induced CNV, the rabbits were instilled with a 1% Mydriacyl eye drop and then anesthetized. After injection of fluorescein sodium salt solution through the ear vein, photographs were taken within 2 min using a fundus camera (TRC-50IX). Retinal CNV confirmation and efficacy evaluation were performed using retinal fluorescence fundus photographs. Image analysis was performed by using ImageJ software.

All animal experiments were carried out according to the guidelines of the Ethics Committee for Protection and Use of Experimental Animals of KNOTUS Co., Ltd. (Incheon, Korea) (Approval number: KNOTUS IACUC 17-KE-171).

### 4.6. Determination of the Therapeutic Efficacies of Ac-RLYE in Comparison with Aflibercept in Animal CNV Model

#### 4.6.1. Mouse CNV Model

In order to determine the therapeutic effects of Ac-RLYE compared with EYLR (reverse-sequence of the RLYE peptide) and RLYE, a laser-induced CNV animal model was prepared as described previously [32]: A bruch membrane between a retinal layer and a choroidal layer was destroyed by irradiating the mouse retina with a diode laser at an intensity of 400 mW and a duration of 50 ms, and then choroidal neovascularization occurred over 14 days. The retinas of 6 week-old male C57BL/6 mice were irradiated with a laser under the above conditions, the formation of a lesion was confirmed through a safety inspection on day 10, and 1 μL of a 1.5 mM EYLR, RLYE, or Ac-RLYE solution was intravitreally injected (6 mice per each treatment group). Here, the same amount of saline was injected into the control group. After 4 days (14 days after the laser irradiation), an experiment for analyzing a therapeutic effect was carried out by extracting the eyeballs of the mice. The extracted eyeballs were fixed in 4% paraformaldehyde for 12 h, and embedded in paraffin. Sections with a thickness of 4 μm were prepared from paraffin tissue, and subjected to H&E staining. Afterward, an image of a section with the largest choroidal neovascular membrane area was taken from each subject, and the area of the choroidal neovascular membrane was quantitatively analyzed using ImageJ program (NIH, Bethesda, Rockville, MD, USA). In the saline-injected control group, the average of the choroidal neovascular areas was represented as 100%, and a therapeutic effect of each treatment group was analyzed.

#### 4.6.2. Rabbit 7-Week CNV Model

After healthy Chinchilla rabbits selected after the acclimation period were instilled with a 1% Midracyl eye drop to the right eye and anesthetized, laser photocoagulation (Elite, Lumenis, San Jose, CA, USA) was performed using a laser with a center wavelength of 532 nm, an incident power of 150 mW, spot size of 200 μm, and pulse duration of 0.1 sec. The intensity of laser irradiation was empirically set in order to maintain the retinal fluorescence intensity in the vehicle treatment group at 7-week after laser irradiation. Six spots were made at about 6 o’clock centered on the optic nerve. 

The test substance was administered once on the 7th day after laser irradiation for induction of active CNV for 1 week, when rabbits were anesthetized and the test substance (50 μL of vehicle, Ac-RLYE or positive control) was injected into the right eye of the animal using a syringe equipped with a 31-gauge needle with the following administration groups (7 rabbits per group): Vehicle (PBS, pH 7.4) control group (G1), Ac-RLYE 100 μg/50 μL/eye treated group (G2), and aflibercept 2000 μg/50 μL/eye treated group (G3).

From the day of laser irradiation to 7, 14, 21, 28, 35, 42, and 49 days after laser induced CNV, the rabbits were instilled with a 1% Mydriacyl eye drop and then anesthetized. After injection of fluorescein sodium salt solution through the ear vein, photographs were taken within 2 min using a fundus camera (TRC-50IX). Retinal CNV confirmation and efficacy evaluation were performed using retinal fluorescence fundus photographs and its image analysis by using ImageJ software.

Fundus photography of retinal angiography was performed at the 42nd day from test substance treatment. Additionally, the eyes were then removed and fixed in 10% formalin solution for 24 h. The cornea, lens, and retina were removed. After washing with PBS, the choroidal epithelial layer and choroid complex (RPE-choroid complex) just remained from fixed eyes. The CNV area was stained with rhodamine conjugated isolectin B4 (IB4, Sigma-Aldrich, St. Louis, MO, USA) for 24 h at 4 °C. After staining, they were washed in PBS, flat-mounted on a glass slide, and then observed with a fluorescence microscope equipped with a digital camera and photographed. The CNV area was measured by ImageJ software.

All animal experiments were carried out according to the guidelines of the Ethics Committee for Protection and Use of Experimental Animals of KNOTUS Co., Ltd. (Incheon, Korea) (Approval number: KNOTUS IACUC 17-KE-246).

### 4.7. Determination of the Intraocular PK Properties of Ac-RLYE

Following intravitreal single administration of Ac-RLYE at 20 μg/50 μL/eye or 100 μg/50 μL/eye in the right eye of 30 male New Zealand White rabbits supplied by Hallym experimental animals (Hwaseong, Korea), the whole blood was collected from jugular vein and then ocular tissues (vitreous body, retina including the choroid, aqueous humor) were sampled from rabbits euthanized at the scheduled sampling points (before Ac-RLYE administration, 2, 6, 12, 24, and 72 h following administration). The isolated plasma and vitreous body were pretreated with stabilizers (bestatin hydrochloride, Cat. No. B8385, 10 μM, Sigma Aldrich Co Ltd., St. Louis, MO, USA) at a ratio of 20 to 1 (e.g., 400 μL plasma or vitreous body +20 μL bestatin) to inactivate the proteinase or peptidase in biological sample and stored with other ocular tissues under −70 °C prior to analysis. All animal experiments were carried out according to the guidelines of the Ethics Committee for Protection and Use of Experimental Animals of KNOTUS Co., Ltd. (Approval number: KNOTUS IACUC 17-KE-196 and 17-KE-282).

Sample treatment and analysis for the quantitative determination of Ac-RLYE in rabbit plasma and ocular tissues were performed by the LC-MS/MS analytical method verified by L2 SCIENCE Co., Ltd. (Ansan, Korea). Study sample analysis was processed with each divided analytical run of the samples, which consisted of system suitability samples [Quality Control Medium (QCM) 5 ng/mL for plasma/retina (including the choroid) and 1 μg/mL for vitreous body/aq. humor, *n* = 5], calibration standards of a blank sample, zero sample, and six or seven calibrator concentrations over a range of 0.1–50 ng/mL [plasma/retina (including the choroid)] and 0.01–10 μg/mL (vitreous body and aqueous humor), quality control samples with three different concentration levels [Quality Control Low (QCL) 0.5 ng/mL, QCM 5 ng/mL, Quality Control High (QCH) 40 ng/mL for plasma/retina (including the choroid) and QCL 0.05 μg/mL, QCM 1 μg/mL, QCH 8 μg/mL for vitreous body and aqueous humor, duplicates] and study samples (plasma/batch). This study samples were requiring the dilution before analysis because the administration dosage was 5 times different between the two groups. Therefore, some of vitreous body and aqueous humor samples were diluted 10 times with blank corresponding to each tissue and analyzed. Dilution integrity was evaluated by 3 replicates after diluting 10 μg/mL QC sample 10 times with blank tissue to bring the concentration within the calibration range.

Acceptance criteria with respect to sample analysis were met on all occasions as follows: No exceed 10% of coefficient of variation for the mean peak area ratio for system suitability, 6–8 concentration levels (excluded blank sample) of calibration standards, within ±15% of nominal value for back-calculated non-zero standard [except for the lower limit of quantification (LLOQ): ±20%], at least 75% of the calibration standards (or minimum of 6 levels) including LLOQ and upper limit of quantification (ULOQ) should meet criteria for calibration curve, not exceed 20% of mean value for analyte and internal standard peak retention time in this batch for retention time, a minimum of 3 different concentration levels (low-, mid-, and high-levels), >6 QCs/batch or >5% of study samples/batch, at least 2/3 (67%) of measured QCs concentration should be within ±15% of the nominal concentration, and 1/2 (50%) of same measured QCs concentration should be within ±15% of the nominal concentration for QCs. Dilution integrity was demonstrated to meet the acceptance criteria, i.e., within ±15%, for accuracy and precision of diluted QC samples.

The calculation of the concentration of Ac-RLYE in the study sample was based on peak area ratios of the analyte to that of internal standard [Tamsulosin hydrochloride (USP, Rockville, MD, USA)]. Weighted linear regression (1/x^2^) was used to fit Ac-RLYE/internal standard peak area ratio versus the theoretical concentration. The concentrations of Ac-RLYE in quality control samples and study samples were calculated through the calibration curves. Software to be used for data processing and calculations were TargetLynx (Waters, Milford, MA, USA) and Microsoft Excel 2013 (Microsoft, Redmond, WA, USA). The PK parameters composed of Area under the concentration-time curve (AUC), maximum concentration (C_max_), time to the maximum concentration (T_max_), terminal elimination half-life (t_1/2_), apparent volume of distribution [Vz(terminal)/F], and clearance (CL/F) were calculated by non-compartmental analysis model using BA Calc 2007 program (supplied by the Ministry of Food and Drug Safety). The systemic exposure to the test substance was determined by calculating AUC from zero to the final sampling time (AUC_last_) using the linear trapezoidal rule. The C_max_ and T_max_ were determined from the analytical data and other parameters such as t_1/2_, Vz/F, and CL/F were used as reference values.

### 4.8. Determination of the TK Properties of Ac-RLYE

Following repeated intravitreal administration of Ac-RLYE at 40, 90, and 200 μg/eye (both eyes; 0, 80, 180, and 400 μg/head, respectively) bi-weekly for 14 weeks in 40 New Zealand White rabbits (5 males and 5 females per each dose group) supplied by Saeronbio Inc. (Uiwang, Korea), the whole blood was collected from cephalic vein on the first day of administration (1st TK) and the 8th administration day (Week 14, 2nd TK) at the scheduled sampling points (before Ac-RLYE administration, 6, 12, 24 h, 2, 3, 5, 7, 10, and 14 days after intravitreal administration). The isolated plasma was pretreated with 20% formic acid (Sigma Aldrich Co Ltd., St. Louis, MO, USA) at a ratio of 5 to 1 (e.g., 180 μL plasma +20 μL 20% formic acid) to inactivate the proteinase or peptidase in biological sample and stored under −45 °C prior to analysis. As Chemon Inc. received approval from AAALAC International for full accreditation in 2010, this study was approved by IACUC of Chemon Inc. (Yongin, Korea) (Approval number: 17-B469).

The Ac-RLYE in rabbit plasma was analyzed with internal standard [Phenformin hydrochloride, BOC science (Shirley, NY, USA)] according to the methods (LC-MS/MS method) which was validated in a test site study [International Scientific Standards Inc. (Chuncheon, Korea), study no.: TG1-17-060]. The pharmacokinetic parameters were calculated using the Phoenix™ WinNonlin^®^ (ver. 6.2, Certara Pharsight, Princeton, NJ, USA) by non-compartmental analysis based on blood concentration curves. Systemic exposure to Ac-RLYE was calculated as area under the curve up to 14 days (AUC_0–14day_) by the linear trapezoidal rule using the test substance concentration at each blood sampling time, and the measured values were used for the peak blood concentration (C_max_) and the time at peak concentration (T_max_). The elimination half-life (t_1/2_) was calculated by the best fitting method from the administration time using the WinNonlin^®^ program.

### 4.9. Comparison of the Amino Acids of the VEGFR-2 Region Targeted by Ac-RLYE in Various Animal Species

As VEGFR-2 is the primary receptor for the VEGF family inducing angiogenesis, and Ac-RLYE has been shown to be a specific inhibitor targeting VEGFR-2 in previous studies [30,31,32,33], we compared the amino acid sequences of the VEGFR-2 region, which is the interaction site for Ac-RLYE, in human (UniProtKB-P35968) and several animal species [mouse (UniProtKB-35918), rat (UniProtKB-O08775), pig (UniProtKB-K7GNF0) and rabbit (UniProtKB-G1SN50)]. The analysis was performed with VEGFR-2 amino acid sequences from the Uniprot database (UniProtKB, www.uniprot.org) (accessed on 11 November 2020) using the program CLC main workbench version 20 (CLC bio, Qiagen, Aarhus, Denmark).

### 4.10. Statistical Analysis

The results of those studies were assumed to be normally distributed. The results of those studies were analyzed by parametric multiple comparison procedures, One-way ANOVA test and additionally analyzed by Student *t*-test in case of the comparison with positive control. When the result of ANOVA was significant, and Dunnett’s multiple comparison test was applied.

All statistical analyses were performed with Prism 5.03 (GraphPad Software Inc., San Diego, CA, USA), and the significance level was judged at *p* < 0.05.

## 5. Conclusions

Based on the VEGF-induced angiogenesis inhibitory effect and short in vivo half-life of RLYE, the N-terminal L-arginine was acetylated (Ac-RLYE) for lead optimization to limit proteolytic degradation. Ac-RLYE has an improved in vivo stability as a pivotal property for new drug development. As Ac-RLYE suppresses retinal endothelial permeability and directly inhibits the VEGF-A/VEGFR-2 signaling pathway in the retina, along with the ability to rapidly reach the retina with minimal systemic exposure and no accumulation in the plasma following single or repeated intravitreal administration, this investigational drug inhibited and efficiently treated retinal neovascularization in animal models (mouse, rat, minipig, and rabbit) of AMD, in which its efficacy was similar or superior for 4–6 weeks at or above the dose of 20 μg/eye (intravitreal injection) compared with conventional therapeutics (ranibizumab and aflibercept). In conclusion, Ac-RLYE is a drug candidate for the treatment of the neovascular (wet) AMD caused by pathological angiogenesis.

## Figures and Tables

**Table 1:** Pharmacokinetic parameters of Ac-RLYE following single intravitreal administration in rabbits.

PK Parameters	Vitreous Body ^1^		Aqueous Humor ^1^		Retina (Including the Choroid) ^2^		Plasma ^3^	
	20 µg/eye	100 µg/eye	20 µg/eye	100 µg/eye	20 µg/eye	100 µg/eye	20 µg/eye	100 µg/eye
AUC(last)	198.78	504.27	23.20	150.47	0.18	34.12	ND ^4^	3.34
C_max_	15.59	42.03	1.94	6.67	0.02	2.62	ND	0.32
T_max_	2.00	2.00	6.00	6.00	2.00	2.00	ND	6.00
CL(inf)/F	0.10	0.20	0.84	0.66	69.96	2.93	ND	2.43
Vz(terminal)/F	1.02	2.04	5.08	8.66	846.31	32.94	ND	302.28
t_1/2_	7.02	7.13	4.20	9.07	8.39	7.80	ND	86.35

^1^ Vitreous and aqueous humor—AUC, μg·hr/mL; C_max_, μg/mL; T_max_ & t_1/2_, hr; CL(inf)/F, L/hr; Vz(terminal)/F, L. ^2^ Retina (including the choroid)—AUC, ng·hr/mg; C_max_, ng/mg; T_max_ & t_1/2_, hr; CL(inf)/F, kg/hr; Vz(terminal)/F, kg. ^3^ Plasma—AUC, ng·hr/mL; C_max_, ng/mL; T_max_ & t_1/2_, hr; CL(inf)/F, L/hr; Vz(terminal)/F, L. ^4^ Not detected (the concentration below the lower limit of quantification).

**Table 2:** Toxicokinetic parameters of Ac-RLYE following repeated intravitreal administration in rabbit.

Male				
Group/ Dose (μg/head/body)	Period	Ac-RLYE Toxicokinetics		
		AUC_0–14day_ (ng *day/mL)	C_max_ (ng/mL)	T_max_ (day)
G2/80	Day 1	0.912	0.657	1.13
	Week 14	0.139	0.246	0.38
G3/180	Day 1	0.651	1.483	0.25
	Week 14	0.309	0.782	0.25
G4/400	Day 1	1.558	3.001	0.25
	Week 14	1.033	2.139	0.25
**Female**				
**Group/** **Dose (μg/head/body)**	**Period**	**Ac-RLYE Toxicokinetics**		
		AUC_0–14day_ (ng *day/mL)	**C_max_ (ng/mL)**	**T_max_ (day)**
G2/80	Day 1	0.156	0.457	0.25
	Week 14	0.021	0.083	0.25
G3/180	Day 1	0.587	1.121	0.25
	Week 14	0.188	0.494	0.25
G4/400	Day 1	1.677	2.796	0.25
	Week 14	0.632	1.555	0.25
**Grand-Mean**				
**Group/** **Dose (μg/head/body)**	**Period**	**Ac-RLYE Toxicokinetics**		
		AUC_0–14day_ (ng *day/mL)	**C_max_ (ng/mL)**	**T_max_ (day)**
G2/80	Day 1	0.534	0.557	0.25
	Week 14	0.080	0.165	0.25
G3/180	Day 1	0.619	1.302	0.25
	Week 14	0.248	0.638	0.25
G4/400	Day 1	1.618	2.899	0.25
	Week 14	0.832	1.847	0.25

## Data Availability

Data is contained within the article or supplementary material.

## Supplementary Materials

Supplementary Materials are available online at https://www.mdpi.com/article/10.3390/ijms22083893/s1.
