# Supplementary material for: Therapeutic Efficacy of a Novel Acetylated Tetrapeptide in Animal Models of Age-Related Macular Degeneration
Source: Int J Mol Sci. 2021 Apr 9;22(8):3893. doi: 10.3390/ijms22083893 (PMC8070582; doi:10.3390/ijms22083893)
Supplement: Supplementary file 1 [file ijms-22-03893-s001.pdf]

Therapeutic Efficacy of a Novel Acetylated Tetrapeptide in Animal Models of Age-Related Macular Degeneration

Hye Cheong Koo [1,†], Yi-Yong Baek [1,†], Jun-Sup Choi [1], Young-Myeong Kim [2], Bokyung Sung [1], Min-Jung Kim [1], Jae Gyu Kim [1] and Ji Chang You [1,3,*]

1 Avixgen Inc., Seoul 06649, Korea; koohj99@avixgen.com (H.C.K.); yybaek@avixgen.com (Y.-Y.B.); mjschoi@daum.net (J.-S.C.); bsung@avixgen.com (B.S.); mjkim208@avixgen.com (M.-J.K.); jagkim@avixgen.com (J.G.K.)
2 Department of Molecular and Cellular Biochemistry, School of Medicine, Kangwon National University, Chuncheon, Gangwon-do 24341, Korea; ymkim@kangwon.ac.kr
3 National Research Laboratory for Molecular Virology, Department of Pathology, School of Medicine, The Catholic University of Korea, Seoul 06591, Korea
* Correspondence: jiyou@catholic.ac.kr; Tel.: +82-2-2258-7312
† These authors are equally contributed.

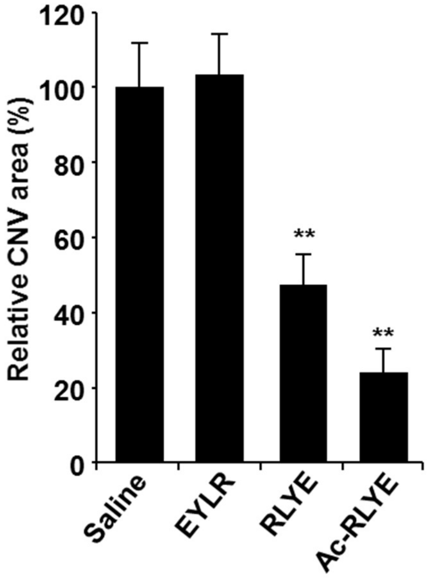

Supplementary Figure S1. The therapeutic effects of the intravitreal administration of EYLR, RLYE, and Ac-RLYE on choroidal neovascularization (CNV) in laser-induced CNV mouse models with treatment of peptide or aflibercept 10 days after laser irradiation to induce active CNV for 10 days. Images of the section with CNV of extracted eyeballs at 4 days after peptide treatment (14 days after the laser irradiation) detected by hematoxylin-eosin (H&E) staining (parts shown by a dotted line represent the boundary of a choroidal neovascularization membrane) were analyzed quantitatively using the ImageJ software for the comparison of area of CNV (n = 6 mice per group). Compared to no change in the occurrence of CNV observed in ELYR (reverse sequence of the RLYE peptide), the intravitreal administration of RLYE and Ac-RLYE significantly reduced the CNV area with the higher effect observed in Ac-RLYE treatment.

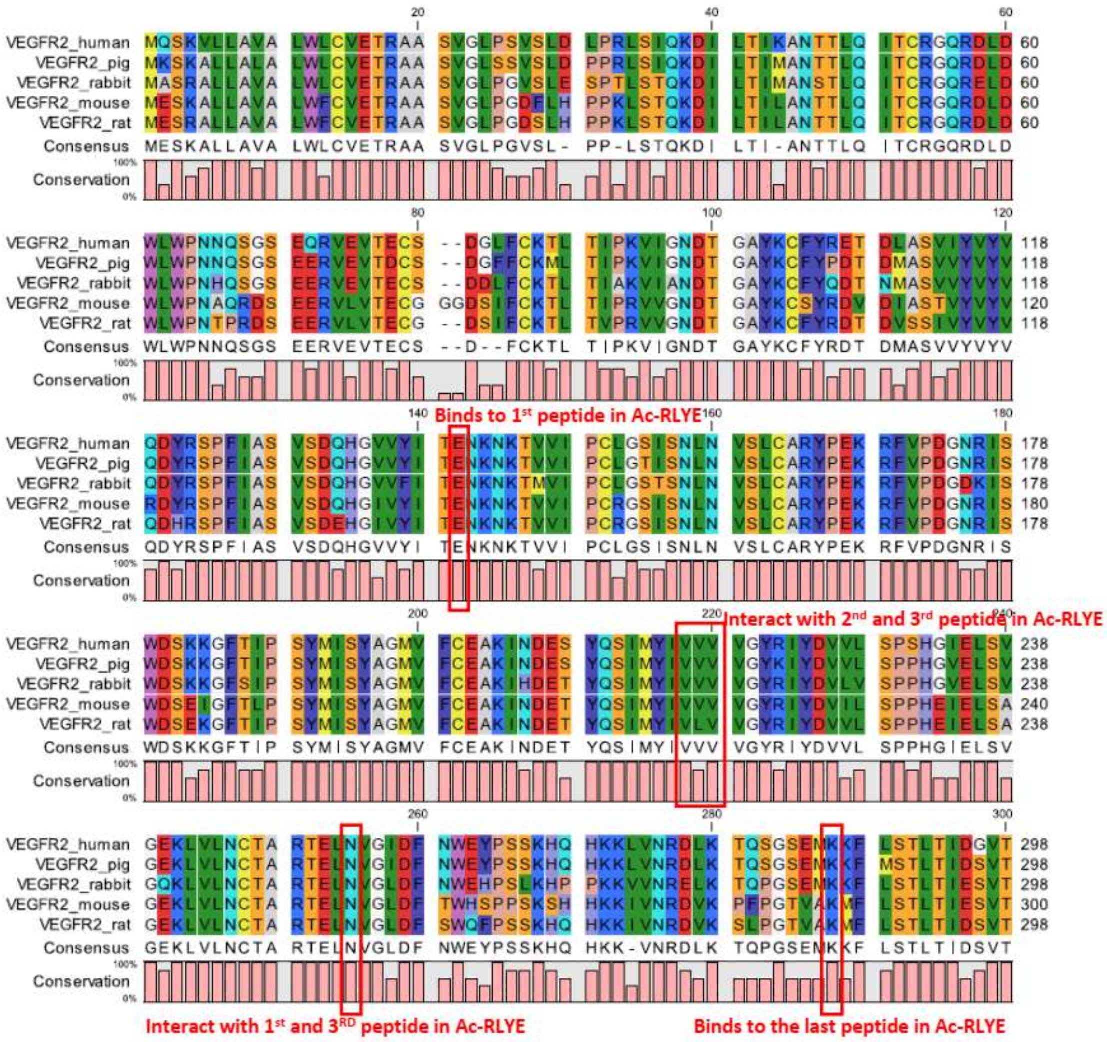

Supplementary Figure S2. Comparison of VEGFR-2 seqences in humans, pig, rabbit, mouse and rat and the site bound by Ac-RLYE. When the amino acid sequences of VEGFR-2 region from Uniprot database (UniProKB) were compared between human and animal species using the program CLC main workbench version 20. The amino acids of the VEGFR-2 targeted by Ac-RLYE are conserved in humans and the animals (mouse, pig, and rabbit) analyzed in this study except one amino acid sequence in rat (valine versus leucine), but both amino acids were hydrophobic amino acids resulting in no negative effect on hydrophobic interaction with the third amino acid of Ac-RLYE, tyrosine.
